# Convulsions in Children

**Published:** 1883-07

**Authors:** 


### Selections.

### Convulsions in Children.

Infantile convulsions must always possess for the practica-physician a keen, almost a fascinating interest. The cases are by no means of equal importance; some may be immediately dangerous to life; some may be merely symptomatic of diseases varying immensely in severity, and some may possess but little significance. As regards the symptom—convulsion—the phenomena are various. The convulsions may be general, and involve all the muscles of the animal life, or they may be limited to a single group of muscles. The symptomatic and the theral peutical diagnosis demand the clearest conception, the greatest fertility of resource, and the utmost promptitude of action.

As above suggested, a convulsion may mean much or little. At the outset, it is best to have as definite a conception as possible of what a convulsion is. That the pons varolii and medulla oblongata are centres of reflex actions has long been known, but it was reserved for Nothnagel to demonstrate the position and define the limits of the "spasm centre." Irritation of this centre induces general convulsions, and this irritation may be direct or reflex, centric or excentric. The results of experimental physiology receive support from pathology. Ladame, in his *Hirngeschwulste*, has formulated this conclusion: When the symptoms of brain tumor exist, if there are convulsions, the tumor is not in the medulla, which may be interpreted as follows:

When a tumor develops in a position to injure the spasm centre, convulsions become impossible because the injured part has lost its power of functionate.

Various causes increase the irritability of the spasm centre. Abnormal irritability may, indeed, be hereditary. It is well known that certain families exhibit the tendency to convulsions, and all the children may experience attacks, or they may be confined to one sex. This tendency may be so strong that infants in the womb are affected, but it is in the first two years of infantile life that the greatest irritability of the spasm centre is found to exist. Beside this tendency, which is inherited, various constitutional states increase the liability to attacks of eclampsia. Rickets has a prominent position as a pathogenetic factor. This state acts, probably, by so increasing the irritability of the centres of reflex action that very slight peripheric irritation sets off the high-strung spasm centre. The state of nutrition of the child is not without influence. When much reduced by long illness, the reflex functions are correspondingly lowered, and hence when, under such circumstances, convulsions occur, it is reasonable to suppose that no peripheric irritation has sufficed, but that some "coarse lesion" of the intra-cranial organs is the cause. Hence it follows that the nutrition of the child suddenly attacked with convulsions has diagnostic value; if the child be fat and healthy, the convulsion is a symptom of some excentric irritation; if weak and emaciated it signifies some centric lesion, notably tuberculous. It is not affirmed that such a rule has no exceptions—only that it has diagnostic value.

It is important to distinguish between eclampsia and epilepsy. Age is an influential element. If a convulsion occur after four or five years of age, if it is over in ten minutes, and no cause can be discovered for it, these constitute good grounds for suspecting epilepsy. If the attack is accompanied by high fever, if albumen can be detected in the urine, or if some acute disease follow, the seizure is one of eclampsia, although the patient may be anywhere from two to ten. Again, the character of the attendant phenomena—the behavior of the convulsion itself—throws strong light on the diagnosis. When the convulsions are limited to the face, to one limb, to one side of the body, it may be concluded that the lesions are intra-cranial. Again, if any part, the seat of convulsion, the face, the limbs, etc., should continue paretic or paralyzed for some days after the seizure or if a squint should continue, or an eyelid droop, or the pupils remain unequal, cerebral lesions probably exist.

The prognosis of convulsions is usually difficult. When arising from intra-cranial lesions, the prospect is gloomy. Such evidences of cerebral mischief as squinting, irregular pupils, coma, etc., are of evil omen. In the convulsions due to uræmic poisoning, the most unfavorable symptoms may be recovered from, but the case wears a less hopeful aspect the more persistent the failure of the urinary excretion. When the breathing continues labored, and there is deep cyanosis, with lividity of the face, and the pulse is very rapid, the case has a most unfavorable appearance. A convulsion at the onset of an acute affection, as scarlet fever, affords no certain indication of the future gravity of the disease, but does illustrate the mobility of the nervous centres. Convulsions occurring toward the close of an acute disease are unfavorable, and often signify that the disease has taken a more serious direction, or that tubercular meningitis has come on. In some children so irritable and mobile is the reflex centre of spasm that but trivial peripheral impressions suffice to bring on convulsions. Amongst other causes, are indigestible food, swollen gums, earache, etc. Such children may have repeated attacks, which, if known, must lessen the gravity of the prognosis. A guarded opinion should be given as respects the future condition of such children, for if convulsions occur readily during the first and even second dentition from slight causes, this is a reason for apprehending the subsequent occurrence of epilepsy. Habit is such an influential factor in determining attacks of nervous diseases that we may well be solicitous regarding its power here.

The treatment of convulsions has an importance determined entirely by the cause of the seizures. Is the attack merely an excited state of the spasm centre from simple peripheric irritation? Has the child eaten some indigestible food? Are there worms, irritating foods, scybala, etc., in the intestinal canal? Is there a stone in the bladder, preputial irritation, or other source of irritation in the genito-urinary tract? Has sufficient urine been passed, and is the urine albuminous? Is an acute disease beginning, and is fever present? Has the child passed through an illness recently, especially of scarlatina or whooping cough? Is the child emaciated? Has the child rickets? The treatment is much influenced by the answers to these questions. Causes of irritation must be at once removed by emetics, purgatives, vermifuges, etc , as required. Then follow the measures to allay the excitability of the spasm centre: bromide of potassium, chloral hydrate, and the inhalation of chloroform. When time presses, the last-mentioned expedient has great value. It is sometimes advised to administer ether instead of chloroform, but this suggestion indicates a failure to appreciate the excitant qualities of the former. Chloroform is well borne by children, and is more effective than ether. Chloral, by the rectum, renders an incontestable service. It is safe in the case of children; it is effective, and, although not so prompt, is more sustained in action than chloroform. Bromide of potassium is most useful after consciousness is restored to prevent future or impending attacks, or to allay the excitement, muscular twitching, etc., which may indicate the onset of convulsions. When swallowing is impossible, bromide may also be given by enema, and it may be combined with chloral for all of the purposes for which the latter is applied. If the surface is cold, the circulation feeble and the skin dry, the child should be put in a bath 100 degrees Fahr. If the same conditions exist in a moist and clammy skin, dry heat should be used, the articles affording it having a temperature of 100 degrees also. If, on the other hand, the temperature of the child is high, reaching 103, 104, or 105 degrees, or more, the cold bath, or the cold wet pack should be employed without hesitation. The character of the bath prescribed will necessarily be affected by the state of the urinary secretion. If it is necessary to compensate in an increased action of the skin for the diminished activity of the kidneys, a warm or vapor bath may be necessary. If albuminuria exists, and the urine is very scanty, the convulsions being distinctly uræmic, a very powerful action of the skin must be secured, and this can be effected by no measure so successfully as by pilocarpin. There can be no doubt of the great good accomplished by this remedy under these circumstances, but any prudent practitioner will avoid inducing a dangerous cardiac depression by the use of large doses. Compensation for the diminished urinary secretion can also be obtained by free catharsis.

We should not fail to mention the remarkable results obtained by Loomis in cases of uræmic convulsions, by the hypodermatic injection of full doses of morphia. Although such treatment has been applied to adults only, and may be inadmissible in children, it throws light on the therapeutical diagnosis. In the simplest cases, almost no treatment may be required. A child has eaten an indigestible meal, has a convulsion, and vomits freely. The stomach emptied, the nervous disturbance ceases, but it is always well in such cases to prescribe some bromide of potassium to allay the reflex irritability and the excitement of the spasm centre. Here, as under all circumstances, no treatment should be instituted that is not the result of a careful survey and a logical deduction from the facts.—*Medical News Editorial.*

